# The engagement of histone lysine methyltransferases with nucleosomes: structural basis, regulatory mechanisms, and therapeutic implications

**DOI:** 10.1093/procel/pwac032

**Published:** 2022-07-27

**Authors:** Yanjing Li, Kexue Ge, Tingting Li, Run Cai, Yong Chen

**Affiliations:** State Key Laboratory of Molecular Biology, National Center for Protein Science Shanghai, Shanghai Institute of Biochemistry and Cell Biology, Center for Excellence in Molecular Cell Science, Chinese Academy of Sciences, 320 Yue Yang Road, Shanghai 200031, China; State Key Laboratory of Bioreactor Engineering, East China University of Science and Technology, Shanghai Collaborative Innovation Center for Biomanufacturing (SCICB), Meilong Road 130, Shanghai 200237, China; State Key Laboratory of Molecular Biology, National Center for Protein Science Shanghai, Shanghai Institute of Biochemistry and Cell Biology, Center for Excellence in Molecular Cell Science, Chinese Academy of Sciences, 320 Yue Yang Road, Shanghai 200031, China; State Key Laboratory of Molecular Biology, National Center for Protein Science Shanghai, Shanghai Institute of Biochemistry and Cell Biology, Center for Excellence in Molecular Cell Science, Chinese Academy of Sciences, 320 Yue Yang Road, Shanghai 200031, China; State Key Laboratory of Molecular Biology, National Center for Protein Science Shanghai, Shanghai Institute of Biochemistry and Cell Biology, Center for Excellence in Molecular Cell Science, Chinese Academy of Sciences, 320 Yue Yang Road, Shanghai 200031, China; State Key Laboratory of Molecular Biology, National Center for Protein Science Shanghai, Shanghai Institute of Biochemistry and Cell Biology, Center for Excellence in Molecular Cell Science, Chinese Academy of Sciences, 320 Yue Yang Road, Shanghai 200031, China

**Keywords:** nucleosome, cryo-EM structures, histone methyltransferases, epigenetics, histone methylation, tumorigenesis

## Abstract

Histone lysine methyltransferases (HKMTs) deposit methyl groups onto lysine residues on histones and play important roles in regulating chromatin structure and gene expression. The structures and functions of HKMTs have been extensively investigated in recent decades, significantly advancing our understanding of the dynamic regulation of histone methylation. Here, we review the recent progress in structural studies of representative HKMTs in complex with nucleosomes (H3K4, H3K27, H3K36, H3K79, and H4K20 methyltransferases), with emphasis on the molecular mechanisms of nucleosome recognition and *trans*-histone crosstalk by these HKMTs. These structural studies inform HKMTs’ roles in tumorigenesis and provide the foundations for developing new therapeutic approaches targeting HKMTs in cancers.

## Introduction

In eukaryotic cells, the nucleosome, which contains four histone proteins (H2A, H2B, H3, H4) tightly wrapped by 147 base pairs of DNA, is the basic unit of chromatin (Onufriev and Schiessel, 2019). Diverse posttranslational modifications (PTMs) usually occur on unstructured histone N- or C-terminal tails (Strahl and Allis, 2000; Kouzarides, 2007). These histone modifications can directly modulate nucleosome structures and epigenetic states and recruit “effector” proteins to chromatin to activate or silence gene expression (Bannister and Kouzarides, 2011). There is increasing evidence that the misregulation of PTMs can contribute to the initiation and development of human cancers (Zhao et al., 2021). Therefore, an in-depth understanding of the regulatory mechanisms of PTMs will guide the identification of new drug targets for human diseases (Chi et al., 2010; Zhao et al., 2021).

Histone methylation is one of the best-characterized PTMs and plays essential roles in many physiological processes, including heterochromatin formation, X-chromosome inactivation, transcriptional regulation, and DNA damage response (Martin and Zhang, 2005). Histone methylation mainly occurs on lysine and arginine residues. Lysine methylation involves transferring one, two, or three methyl groups from the donor *S*-adenosylmethionine (AdoMet) to the ε-nitrogen of the lysine residue. There are six well-studied lysine methylation sites in mammalian cells: K4, K9, K27, K36, and K79 on H3 and K20 on H4. Different methylation sites and different methylation levels have distinct biological outcomes, and their combinations significantly increase the complexity of “histone codes” in regulating gene expression (Black et al., 2012; Mohan et al., 2012). In general, tri-methylation of H3K4, H3K36, and H3K79 is predominantly associated with transcriptional activation, whereas tri-methylation of H3K9, H3K27, and H4K20 is usually correlated with transcriptional repression or silencing (Black et al., 2012; Mohan et al., 2012).

Lysine methylation marks are deposited by histone lysine methyltransferases (HKMTs). All HKMTs, with one exception (Dot1-family methyltransferases), contain a conserved enzymatic SET [SU(VAR)3-9, E(Z), and TRX] domain initially identified in su(var)3-9, enhancer-of-zeste, and trithorax proteins (Mohan et al., 2012). The enzymatic activities of HKMTs are tightly regulated by many *cis*- and *trans*-factors. The catalytic and regulatory mechanisms have been uncovered by biochemical and structural studies of these HKMTs, which have been extensively reviewed (Worden and Wolberger, 2019; Janna et al., 2020; Glancy et al., 2021; Liu, 2021; Zhai and Brownell, 2021; Lam et al., 2022). Here, we focus on recent progress in structural studies of representative HKMTs in complexes with nucleosomes, a more physiological substrate than the histone peptide. This review summarizes the molecular mechanisms of nucleosome recognition and *trans*-histone crosstalk regulation by these HKMTs. These structural studies provide insights into how disease-associated mutations affect HKMT functions and lay the foundations for drug design for some HKMT-related diseases.

## Structures of HKMTs in complex with nucleosomes

**Figure 1.**
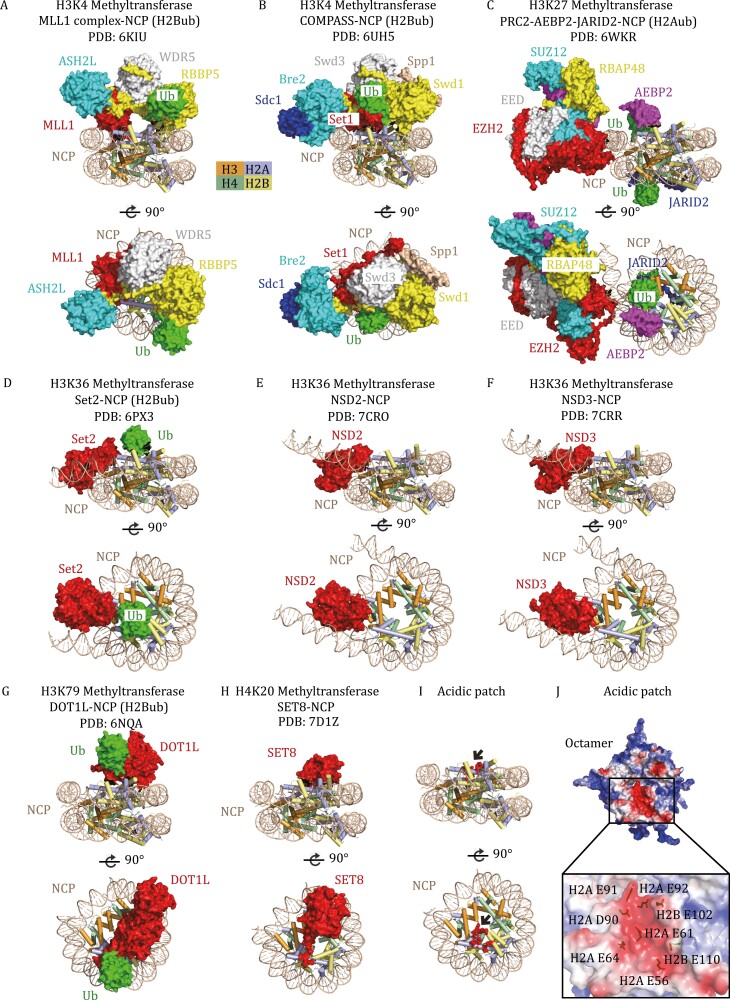
Representative HKMTs in complex with nucleosomes. (A) Cryo-EM structure of the human MLL1 complex with an H2B K120ub-modified NCP (PDB: 6KIU), shown from two orthogonal views. The catalytic SET domain of MLL1 (red) forms a multimeric core complex with ASH2L (blue), WDR5 (white), and RBBP5 (yellow) to achieve specific activities. MLL1, ASH2L, WDR5, RBBP5, and Ub (green) are shown in surface representation, and the structure of NCP is shown in a cartoon diagram. (B) Cryo-EM structure of *K. lactis* COMPASS bound to H2Bub NCP (PDB: 6UH5). The extended catalytic module of the COMPASS complex is composed of Set1 (MLL-homolog), Swd3 (WDR5 homolog), Swd1 (RBBP5 homolog), Bre2 (ASH2L homolog), Sdc1 (DPY30 homolog) and Spp1 (CFP1 homolog). (C) Cryo-EM structure of human polycomb repressive complex 2.2 (PRC2.2) in complex with H2AK119ub-modified NCP (PDB: 6WKR). The PRC2.2 complex comprises PRC2 core proteins (EZH2, SUZ12, EED, RBAP46/48) and cofactors JARID2 and AEBP2. (D) Cryo-EM structure of *Chaetomium thermophilum* Set2 bound to H2Bub NCP (PDB: 6PX3). (E) Cryo-EM structure of the human NSD2-NCP complex (PDB: 7CRO). (F) Cryo-EM structure of the human NSD3-NCP complex (PDB: 7CRR). (G) Cryo-EM structure of human DOT1L bound to H2Bub NCP (PDB: 6NQA). (H) Cryo-EM structure of the human SET8–NCP complex (PDB: 7D1Z). (I) The H2A–H2B acidic patch on the NCP. The acidic patch is formed by E56, E61, E64, D90, E91, and E92 of H2A and E102 and E110 of H2B, which are shown as red spheres and indicated by black arrows. The structure of NCP is shown in a cartoon diagram. (J) Electrostatic potential view of the octamer surface (positive potential, blue; negative potential, red). The eight glutamate/aspartate residues in the H2A-H2B acidic patch are shown in stick models.

With the rapid development of single-particle cryo-electron microscopy (cryo-EM), more than forty structures of HKMTs in complex with nucleosome core particles (NCPs) have recently been solved (Table 1), including methyltransferases for H3K4, H3K27, H3K36, H3K79, and H4K20 methylation. The structures of H3K9 methyltransferases with nucleosomes have yet to be determined. Although these HKMTs share the conserved catalytic domain and exhibit similar catalytic mechanisms when using histone peptides as substrates, they engage nucleosomes in diverse modes (Fig. 1A–H). A common HKMT-binding platform on nucleosomes is the acidic patch formed by a group of aspartate and glutamate residues from H2A and H2B (Fig. 1I and 1J) (Luger et al., 1997; Kalashnikova et al., 2013; Zhou et al., 2019). Nevertheless, each HKMT uses a unique structural element to bind the acidic patch (Fig. 1A–H), further highlighting the intricacies of nucleosome recognition by HKMTs.

**Table 1.** HKMTs in complex with nucleosomes.

Methylation site	Protein	PDB	References
H3K4 Methyltransferases	MLL1 complex-nucleosome	6W5I; 6W5M; 6W5N; 6PWV; 6KIX; 6KIZ;7MBN; 7MBM	Park et al., (2019); Xue et al., (2019); Lee et al., (2021) and Ayoub et al., (2022)
	MLL1 complex-nucleosome (H2Bub)	6KIU; 6KIV	Xue et al., (2019)
	MLL3 complex-nucleosome (H2Bub)	6KIW	Xue et al., (2019)
	RBBP5-nucleosome	6PWX	Park et al., (2019)
	MLL1-RBBP5-WDR5-nucleosome	6PWW	Park et al., (2019)
	COMPASS-nucleosome	6UGM	Hsu et al., (2019)
	COMPASS-nucleosome (H2Bub)	6UH5; 6VEN	Hsu et al., (2019) and Worden et al., (2020)
H3K27 Methyltransferases	PRC2:EZH1-nucleosome (H2Aub), PRC2:EZH1	7KSR; 7KTP	Grau et al., (2021)
	PRC2:EZH1-nucleosome (H2Aub), nucleosome	7KTQ	Grau et al., (2021)
	PRC2:EZH1-AEBP2-JARID2	7KSO	Grau et al., (2021)
	PRC2:EZH2-AEBP2-JARID2-nucleosome (H2Aub)	6WKR	Kasinath et al., (2021)
	PRC2:EZH2-nucleosome	7AT8	Finogenova et al., (2020)
H3K36 Methyltransferases	Set2-nucleosome (H2Bub)	6PX3; 6NZO	Bilokapic and Halic, (2019)
	Set2/SETD2-nucleosome	7EA5; 7EA8	Liu et al., (2021)
	NSD2-nucleosome	7CRO	Li et al., (2021a)
	NSD3-nucleosome	7CRP; 7CRQ; 7CRR	Li et al., (2021a)
H3K79 Methyltransferases	DOT1L-nucleosome (H2Bub)	6NQA; 6NOG; 6NJ9; 6NN6; 6JMA; 6O96; 6J99	Anderson et al., (2019); Jang et al., (2019); Valencia-Sánchez et al., (2019); Worden et al., (2019) and Yao et al., (2019)
	DOT1L-nucleosome	6JM9	Jang et al., (2019)
	Dot1-nucleosome (H2Bub)	7K6P	Valencia-Sánchez et al., (2021)
	Dot1-nucleosome (H2Bub and H4K16ac)	7K6Q	Valencia-Sánchez et al., (2021)
H4K20 Methyltransferases	SET8-nucleosome	7D1Z	Ho et al., (2021)
	SET8-nucleosome (CENP-A)	7D20	Ho et al., (2021)

MLL complex: MLL-RBBP5-WDR5-ASH2L (-DPY30); COMPASS: Set1-Swd3-Swd1-Spp1-Bre2-Sdc1; PRC2: EZH1: EZH1-EED-SUZ12-RBAP48; PRC2: EZH2: EZH2-EED-SUZ12-RBAP48

### H3K4 methyltransferases

Methylation of H3K4 is predominantly mediated by MLL (also named KMT2 or SET1) family methyltransferases, which are conserved from yeast to mammals. There is only one member (Set1) in *Saccharomyces cerevisiae*, while mammalian MLL family proteins contain six members, including MLL1 (KMT2A), MLL2 (KMT2B), MLL3 (KMT2C), MLL4 (KMT2D), SETD1A (KMT2F), and SETD1B (KMT2G). MLL-family proteins can methylate H3K4 through their C-terminal catalytic SET domain, but their intrinsic HKMT activities are extremely low (Dou et al., 2006). The HKMT activity of MLL can be significantly stimulated by four conserved core subunits: WD repeat-containing protein 5 (WDR5), retinoblastoma-binding protein 5 (RBBP5), absent, small or homeotic 2-like protein (ASH2L), and dumpy-30 (DPY30) (Dou et al., 2006; Patel et al., 2009). Structural studies of MLL complexes from different species, either in free form or in complex with histone peptides, reveal the conserved assembly mechanism and the important regulatory elements to maintain the optimal HKMT activity of the MLL core complex (Li et al., 2016; Hsu et al., 2018; Qu et al., 2018), but these structures failed to elucidate how MLL complexes methylate nucleosomes and how their activities are regulated by *trans*-histone crosstalk.

**Figure 2.**
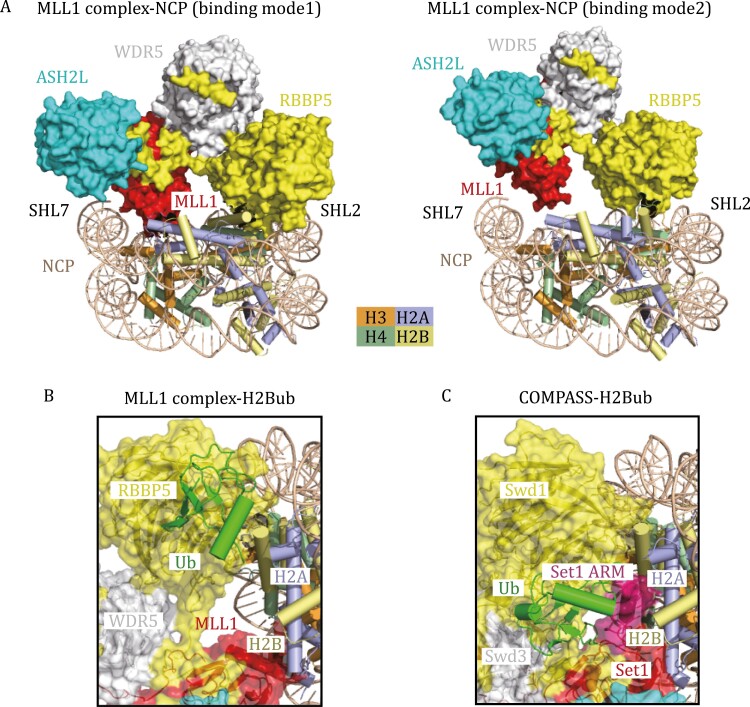
Cryo-EM structures of H3K4 methyltransferases in complex with nucleosomes. (A) Two different binding modes of the MLL1 complex on the unmodified NCP. In binding model 1 (PDB: 6KIX), the MLL1 complex lies on the disc face of the NCP, positioning the MLL1 SET domain to SHL7 DNA and histone H2A. In binding model 2 (PDB: 6KIZ), the MLL1 SET domain moves away from histone H2A, indicating the inherent flexibility of the MLL1 complex association with NCP. (B) Cryo-EM structure of the human MLL1 complex with H2B K120ub-modified NCP (PDB: 6KIU) in a cartoon diagram. The transparent surface of the MLL1 complex is shown. Ubiquitin makes direct contact with RBBP5 and traps the MLL1–NCP complex in binding mode 1. (C) Cryo-EM structure of *K. lactis* COMPASS bound to H2Bub NCP (PDB: 6UH5) in a cartoon diagram. Ubiquitin forms complicated interaction networks with Swd1 (RBBP5) and Set1, further stabilizing the ARM (colored in purple) helix of Set1.

More recently, several cryo-EM structures of MLL1, MLL3, and yeast MLL-homolog COMPASS (complex proteins associated with Set1) in complex with NCP were reported (Hsu et al., 2019; Park et al., 2019; Xue et al., 2019; Worden et al., 2020; Lee et al., 2021), shedding light on how MLL complexes recognize nucleosomes. These cryo-EM structures manifest conserved binding interfaces between NCP and MLL-family complexes. The RBBP5 WD40 domain, ASH2L pre-SPRY/SPRY domain, and MLL SET domain are the major NCP-recognizing regions (Park et al., 2019; Xue et al., 2019; Lee et al., 2021). Specifically, the Y-shaped MLL1 core complex lies on the disc face of the nucleosome through the recognition of the H2B–H4 cleft and DNA superhelix (SHL) 2 by the RBBP5 WD40 domain and SHL7 DNA by ASH2L (Fig. 2A). Additionally, the MLL1 SET domain lies close to SHL7 DNA and makes direct contact with the C-terminal helical region of histone H2A (Fig. 2A), thereby facilitating H3 tail orientation to the catalytic SET domain (Xue et al., 2019).

Notably, the MLL1 complex could bind unmodified NCP in multiple binding modes (mode 1 and mode 2 in Fig. 2A), indicating the inherent flexibility of the MLL1 complex association with NCP and explaining the relatively low activity of the MLL1 complex on unmodified NCP (Xue et al., 2019). Methylation of H3K4 by MLL family proteins or COMPASS can be stimulated by monoubiquitination of histone H2B K120 (or K123 in yeast H2B) (Sun and Allis, 2002; Kim et al., 2009, 2013; Wu et al., 2013). Structural analysis of the MLL1 complex with H2B K120ub-modified NCP (H2BubNCP) reveals that the binding of RBBP5 to ubiquitin reduces the dynamics of the MLL1 complex and traps MLL1 in specific binding mode 1 on NCP, which ensures the binding of the H3 tail in the catalytic pocket of MLL1 (Fig. 2B) (Xue et al., 2019).

H2Bub has a more profound activity-stimulating effect on the yeast COMPASS complex than on the human MLL complex, consistent with the more complicated interaction networks between COMPASS and ubiquitin, as revealed by the cryo-EM structures of yeast COMPASS-H2BubNCP (Hsu et al., 2019; Worden et al., 2020). In addition to the primary contact between ubiquitin and Swd1 (the RBBP5 homolog), ubiquitin can also interact with the N-terminal region of Set1_SET_ and stabilize the arginine-rich motif (ARM) helix of Set1 by fastening it to the nucleosomal acidic patch (Fig. 2C) (Hsu et al., 2019). In this case, ubiquitin enhances enzyme activity by releasing ARM-mediated autoinhibition (Hsu et al., 2019). Interestingly, the ARM helix is unique to yeast Set1 and its mammalian orthologs SET1A and SET1B but not to MLL1–4, indicating that ubiquitin may have distinct regulatory roles in different MLL family proteins. Further structural studies of SET1A-ubNCP and SET1B-ubNCP complexes will be needed to confirm the universal crosstalk mechanism between H3K4 methylation and H2B ubiquitination.

### H3K27 methyltransferases

Polycomb repressive complex 2 (PRC2) is essential for maintaining transcriptional repression and cellular identity (Schuettengruber et al., 2017). The core PRC2 complex, containing enhancer-of-zeste homolog 1/2 (EZH1/2), suppressor of zeste 12 (SUZ12), embryonic ectoderm development (EED), and retinoblastoma-associated protein 46/48 (RBAP46/48), catalyzes the mono-, di- and tri-methylation of H3K27 (Cao et al., 2002; Margueron and Reinberg, 2011). According to the different accessory proteins, PRC2 is further classified into two subtypes referred to as PRC2.1 (containing PHF1, MTF2, PHF19, or EPOP, or PALI1/2) and PRC2.2 (containing JARID2 and AEBP2) (Beringer et al., 2016; Hauri et al., 2016; Liefke et al., 2016; Conway et al., 2018).

Similar to the MLL-family methyltransferases, the intrinsic activity of the catalytic subunit EZH2 is low, and optimal activity is achieved on the core trimeric complex composed of EZH2-EED-SUZ12. H3K27me3-containing peptides can further stimulate the enzymatic activity of the PRC2 core complex. Mechanistic insights into the complex assembly and allosteric activation were elucidated by the crystal structures of the core PRC2 complexes containing EZH2, EED, and SUZ12 from *Chaetomium thermophilum* and humans (Jiao and Liu, 2015; Brooun et al., 2016; Justin et al., 2016). These structural studies reveal that the H3K27me3-containing stimulation peptide is sensed by EED and the EZH2 stimulation-responsive motif (SRM). Upon H3K27me3 binding, the EZH2 SRM undergoes a disorder-to-helix conformational change and then allosterically induces the rotation of EZH2 SET-insertion motif to open the substrate-binding channel, resulting in stimulated activity (Jiao and Liu, 2015).

**Figure 3.**
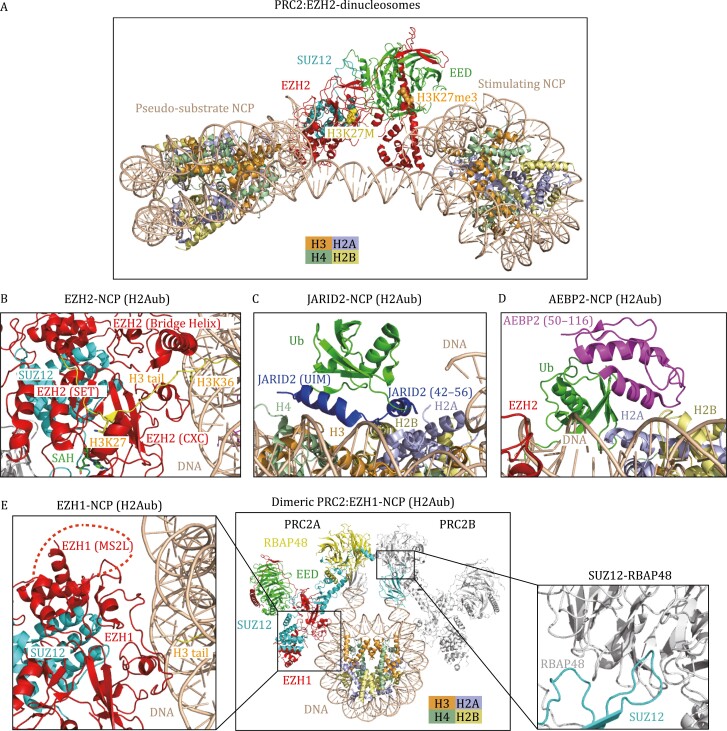
Cryo-EM structures of H3K27 methyltransferases in complex with nucleosomes. (A) Structural model of PRC2 (EZH2-EED-SUZ12) in complex with two functionally distinct nucleosomes (Poepsel et al., 2018). The H3 tails of the pseudosubstrate NCP (H3K27M) and the stimulating NCP (H3K27me3) interact with the SET domain of EZH2 and the EED subunit, respectively. H3K27M and H3K27me3 are shown as spheres. (B–D) Cryo-EM structure of the human PRC2.2 (EZH2-EED-SUZ12-JARID2-AEBP2) complex bound to H2AK119ub-modified NCP (PDB: 6WKR) in a cartoon diagram. (B) The interface between EZH2 and H2Aub NCP. The CXC domain and bridge helix of EZH2 ensure the correct alignment of the H3 tail. H3K36 is located at the binding interface between the EZH2 CXC domain and DNA. H3K27, H3K36, and SAH are shown as stick models. (C) The interface between JARID2 and H2Aub NCP. JARID2 recognizes ubiquitin and the H2A–H2B acidic patch through the UIM domain and JARID2 42–56, respectively; (D) the interface between AEBP2 and H2Aub NCP. AEBP2 interacts with ubiquitin and an H2A–H2B surface through two C2H2 zinc fingers (amino acids 50–116). (E) Cryo-EM structure of dimeric PRC2 (EZH1-EED-SUZ12) in complex with H3K27M and H2AK119ub-modified NCP. The structure is derived from three PDBs (7KSR, 7KTP, 7KTQ). The MS2L in EZH1 is structurally invisible and indicated by a red dashed arc. The interaction between SUZ12 and RBAP48 enables the dimerization of PRC2.

In the past 4 years, the cryo-EM structures of PRC2 in complex with nucleosomes have provided new insights into how the PRC2.1 or PRC2.2 complex engages its native substrate, how regulatory proteins modulate PRC2 activity, and how other histone modifications influence PRC2 activity (Poepsel et al., 2018; Finogenova et al., 2020; Grau et al., 2021; Kasinath et al., 2021). The first cryo-EM structure of the PRC2-nucleosome captured a single PRC2 complex simultaneously bound to a bifunctional dinucleosome composed of a stimulating nucleosome (H3K27me3) and a pseudosubstrate nucleosome (H3K27M) (Poepsel et al., 2018) (Fig. 3A). The positively charged patches of EED and the SANT-binding domain (SBD) of EZH2 interact with the stimulating nucleosomal DNA, while the EZH2 CXC domain binds to the pseudosubstrate nucleosomal DNA, which form the dominant PRC2-nucleosome interface (Poepsel et al., 2018). The H3K27me3 in the stimulating nucleosome binds to EED. As a result, the H3K27me3 signal from the neighboring nucleosome could be transmitted into the substrate nucleosome and allosterically stimulate H3K27me3 methylation, supporting the previous finding that PRC2 had much higher enzymatic activity on dinucleosomes than on mononucleosomes (Yuan et al., 2012). These structural and biochemical studies highlight the crucial roles of PRC2 in H3K27me3 spreading on chromatin and epigenetic memory of repression. Interestingly, PRC2 can also endure the geometrical alterations imposed by various lengths of linker DNA within reconstituted dinucleosomes (30 bp, 35 bp and 40 bp of linker DNA) (Poepsel et al., 2018), indicating the possibility of PRC2 engagement in a diverse chromatin environment.

Recent studies have suggested extensive crosstalk between PRC2-mediated H3K27 methylation and other histone modifications, including H2AK119ub, H3K4me3, and H3K36me2/3 (Schmitges et al., 2011; Yuan et al., 2011; Voigt et al., 2012; Kalb et al., 2014). The cryo-EM structure of PRC2.2 in a complex with an H2AK119ub1-modified NCP highlighted the regulatory roles of JARID2 and AEBP2 in recognizing H2AK119ub and stimulating PRC2 activity (Kasinath et al., 2021). In addition to the contacts of EZH2 with the nucleosomal DNA and the H3 tail seen in the previous PRC2-nucleosome structure (Fig. 3A and 3B), JARID2 and AEBP2 provide additional anchoring sites for the ubiquitinated nucleosome (Kasinath et al., 2021). The ubiquitin interacting motif (UIM) of JARID2 interacts with the hydrophobic patch on ubiquitin (I44), and the JARID2 fragment (residues 42–56) binds the H2A-H2B acidic patch (Kasinath et al., 2021) (Fig. 3C). AEBP2 uses its two C2H2 zinc fingers to recognize ubiquitin and H2A-H2B on the other side of the nucleosome (Kasinath et al., 2021) (Fig. 3D). Interestingly, this structure reveals a disordered K/R-rich region of EZH2 (aa 497–511), which can form an alpha helix (EZH2 bridge helix) upon binding to the nucleosome (Kasinath et al., 2021) (Fig. 3B). The EZH2 bridge helix is sandwiched between the nucleosomal DNA, the SET domain, and the H3 tail, thereby ensuring the correct coordination of the H3 tail in the active site of the EZH2 SET domain. In addition, the structures of PRC2.2 bound to the H2A K119ub-modified nucleosome and PRC2.1 (PHF1–PRC2 complex) bound to a dinucleosome indicate that H3K36 is located at the binding interface between the EZH2 CXC domain and DNA, and methylation of H3K36 would presumably be less fit in the interface and thereby inhibit PRC2 activity (Finogenova et al., 2020; Kasinath et al., 2021), which nicely explains the previous findings that PRC2 could sense H3K36me3 marks to suppress its activity (Schmitges et al., 2011; Yuan et al., 2011; Voigt et al., 2012).

EZH1 is a paralog of EZH2 and has different functions from EZH2, but the structural elements that distinguish EZH1 from EZH2 remain elusive. Recently, a cryo-EM structure of a dimeric PRC2:EZH1 complex bound to a mononucleosome containing H3K27M and H2AK119ub was reported (Fig. 3E), providing insights into the structural difference between EZH1 and EZH2-containing PRC2 complexes (Grau et al., 2021). The primary interaction interfaces between the EZH1-containing PRC2 complex and the nucleosome are similar to those between the EZH2-containing complex and the nucleosome (Fig. 3E). Notably, a loop between the MCSS (motif connecting SANT1L and SANT2L) and SANT2L domains, referred to as the MCSS/SANT2L loop (MS2L), has significant sequence divergence between EZH1 and EZH2. The MS2L loop is near the nucleosomal DNA and might be involved in nucleosome binding (Fig. 3E). Thus, the divergent MS2L may partially explain the disparity of nucleosome binding activity and methyltransferase activity between the EZH1 complex and EZH2 complex. Another interesting feature of the EZH1 complex is the dimerization of the EZH1 complex on one nucleosome. The dimerization of the EZH1 complex is mediated by the domain swap between SUZ12 and RBAP48 (RBBP4) (Fig. 3E) (Grau et al., 2021). Remarkably, the dimeric EZH1 complex is fourfold more effective at promoting chromatin compaction than the monomeric EZH1 complex, although the dimeric and monomeric complexes show similar nucleosome binding affinities and methyltransferase activities. The dimerized EZH1 complex also compacts chromatin more efficiently than the EZH2 complex, suggesting that the dimerization of EZH1-containing PRC2 could promote multivalent interactions within heterochromatin to facilitate compaction. Together, these results reveal the structural variations responsible for the functional distinctions between EZH1- and EZH2-containing PRC2 complexes (Grau et al., 2021).

### H3K36 methyltransferases

Set2 is the sole H3K36 methyltransferase in yeast (Strahl et al., 2002), whereas there are at least seven H3K36 methyltransferases in mammalian cells, including SETD2 (KMT3A), NSD1 (KMT3B), NSD2 (WHSC1), NSD3 (WHSC1L1), SMYD2 (KMT3C), SETMAR, and ASH1L (KMT2H) (Wagner and Carpenter, 2012). Cryo-EM structures of yeast Set2, human SETD2, NSD2, and NSD3 in complex with NCPs reveal conserved structural features for nucleosome binding by H3K36 methyltransferases (Bilokapic and Halic, 2019; Li et al., 2021a; Liu et al., 2021), suggesting a common substrate recognition and activity regulation mechanism involved in H3K36 methylation.

Human SETD2 or yeast Set2 binding with wild-type NCP is rather dynamic, reflected by the invisible density for SETD2 or Set2 in the cryo-EM reconstruction of the SETD2-NCP^WT^ or Set2-NCP^WT^ complex (Bilokapic and Halic, 2019; Liu et al., 2021). The onco-histone mutation H3K36M stabilizes the association of SETD2/Set2 with NCP, enabling the visualization of SETD2/Set2 density in cryo-EM reconstruction (Bilokapic and Halic, 2019; Liu et al., 2021). Set2 binding with NCP induces unwrapping DNA at the entry and exit regions, resulting in the direct interaction between Set2 and nucleosomal DNA (Bilokapic and Halic, 2019). The Set2–DNA interaction induces the rearrangement of the post-SET motif that partially occupies the active site in the apo state (Yang et al., 2016; Bilokapic and Halic, 2019), thus releasing Set2 from the autoinhibitory state. Set2 also makes both hydrophobic and charged interactions with H3 αN, the H3 tail, and the H2A C-terminal tail but does not contact the acidic patch, which is unique for H3K36-family methyltransferases (Bilokapic and Halic, 2019; Liu et al., 2021). The specific histone and DNA interactions align the K36 residue into the active pocket of Set2, explaining the substrate preference of nucleosomes over histone peptides (Du et al., 2008).

The cryo-EM structures of Set2 or SETD2 in complex with NCP also provide insights into the crosstalk between histone ubiquitination and H3K36 methylation. Ubiquitination of H2B K120 enhances the activity of Set2 by generating an additional binding interface between ubiquitin and the Set2 AWS (associated with SET) domain to anchor Set2 on nucleosomes (Bilokapic and Halic, 2019). In contrast, Set2 makes extensive contact with the H2A C-terminal tail, and ubiquitination of H2A K119 presumably disrupts this interface, which is consistent with the finding that ubiquitination of H2A K119 inhibits HKMT activity of H3K36 methyltransferases (Yuan et al., 2013).

The NSD family of methyltransferases plays a crucial role in chromatin regulation and is closely linked to oncogenesis (Bennett et al., 2017). The recently reported cryo-EM structures of NSD2-NCP and NSD3-NCP showed striking similarity to Set2-NCP or SETD2-NCP structures, which share conserved DNA-enzyme and histone-enzyme binding interfaces (Li et al., 2021a). Specifically, the binding of NSD3 to NCP induces the unwrapping of nucleosomal DNA from the entry and exit sites, thereby ensuring NSD3 insertion between unwrapped DNA and the histone core. NSD3 makes extensive contacts with nucleosomal DNA through its N-terminal loop, SET, and post-SET domains (Li et al., 2021a). Complementing the NSD3–DNA interactions, NSD3 also mediates some critical contacts with histones, including the interactions of the NSD3 AWS domain with H3 αN and the H2A C-terminal tail. These structures clearly explain why NSD family methyltransferases methylate H3K36 only in the context of nucleosome substrates.

### H3K79 methyltransferases

Dot1 (disruptor of telomeric silencing 1) or DOT1L (Dot1-like) is the only known methyltransferase that can catalyze mono-, di- and tri-methylation of H3K79 in yeast or mammals, respectively (Feng et al., 2002; Lacoste et al., 2002; Ng et al., 2002a; van Leeuwen et al., 2002). Unlike other histone lysine methyltransferases, DOT1L does not have the characteristic SET domain. DOT1L belongs to the seven-β-strand methyltransferase family and shares structural characteristics of DNA methyltransferases and protein arginine methyltransferases (Min et al., 2003; Petrossian and Clarke, 2011). While most known histone lysine methylation sites are found in the unstructured N-terminal tails of histones, H3K79 is located in the structured histone globular region. Therefore, Dot1 or DOT1L has several unique structural features and biochemical properties that distinguish it from other lysine methyltransferases.

**Figure 4.**
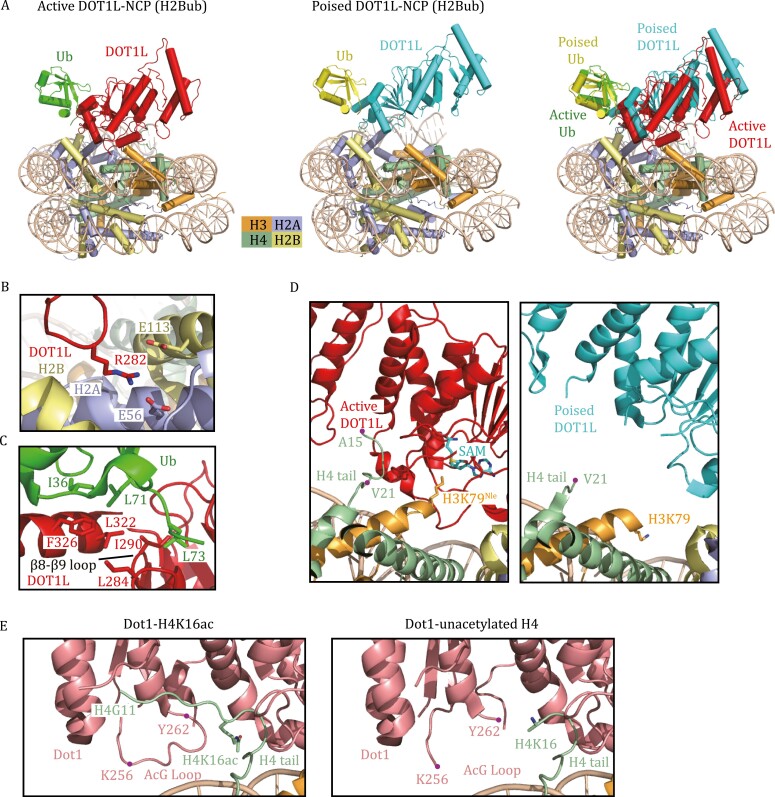
Cryo-EM structures of H3K79 methyltransferases in complex with nucleosomes. (A) Cryo-EM structures of human DOT1L bound to H2Bub NCP in the active state (PDB: 6NJ9) and the poised state (PDB: 6NOG). The active state was trapped by using H3K79^Nle^- and H2Bub-modified NCP. The superimposed structures of DOT1L-H2Bub NCP in the poised and active states show that the catalytic domain of DOT1L in the active state moves toward the nucleosome disc. Nle stands for norleucine. (B) Detailed view of the interactions between DOT1L and the nucleosomal acidic patch. Key residues at the interface are shown as stick models. (C) Detailed view of the interactions between DOT1L and ubiquitin. Key residues at the interface are shown as stick models. (D) Close-up view of the catalytic domain of DOT1L interacting with H3–H4. The H4 tail (16–19) binds DOT1L to position the catalytic site of DOT1L over H3K79 in the active state (left). This H4 tail (16–19) in the poised state is structurally invisible (right). H3K79, H3K79^Nle^, and AdoMet are shown as stick models. (E) Close-up view of the yeast Dot1 interacting with the H4 tail in the Dot1-H4K16ac structure and Dot1-unacetylated H4 structure (PDB: 7K6Q, 7K6P). The H4 tail (G11-K16ac) and the AcG loop 256–262 are clearly visible in the Dot1-H4K16ac structure.

Dot1 or DOT1L methylates only nucleosome substrates but not histone octamers or H3 (van Leeuwen et al., 2002). Five groups independently reported the cryo-EM structures of DOT1L bound to nucleosomes and collectively revealed the molecular mechanism of nucleosome recognition by DOT1L (Anderson et al., 2019; Jang et al., 2019; Valencia-Sánchez et al., 2019; Worden et al., 2019; Yao et al., 2019). The cryo-EM structures of DOT1L in complex with unmodified NCP showed a highly dynamic association, as evidenced by the weak and blurred cryo-EM density maps of DOT1L. H2B K120ub could rigidly constrain the mobility of DOT1L and stabilize the conformation of the enzyme on the nucleosome, consistent with the observation that H2Bub stimulates the catalytic activity of DOT1L (Briggs et al., 2002; Ng et al., 2002b; McGinty et al., 2008). In both DOT1L-NCP and DOT1L-ubNCP structures, DOT1L is anchored to the nucleosomal acidic patch through the interactions mediated by residue R282 (Fig. 4B), confirming that this is the most critical anchor point of DOT1L on the nucleosome (Anderson et al., 2019; Jang et al., 2019; Valencia-Sánchez et al., 2019; Worden et al., 2019; Yao et al., 2019). In the DOT1L-ubNCP structure, the hydrophobic patch comprised of ubiquitin residues I36, L71, and L73 makes contact with the DOT1L C-terminal helix (320–330) and a loop between β8 and β9 (284–290) (Fig. 4C), thereby stabilizing DOT1L association with NCP.

Notably, the structure of the DOT1L-ubNCP (H2B K120ub) complex is often captured in a poised state, in which the active site of DOT1L is too far from H3K79 for efficient catalysis (Fig. 4A and 4D). Using an H2BK120ub nucleosome containing H3K79^Nle^ (Nle stands for norleucine, a nonnative amino acid that has been shown to improve methyltransferase–substrate interactions), the Wolberger laboratory reported the structure of DOT1L bound to ubNCP in the active state (Worden et al., 2019). Compared to the poised state, the catalytic domain of DOT1L in the active state is pivoted toward the nucleosome face (Fig. 4A) (Worden et al., 2019). A stretch of basic residues (_16_KRHR_19_) in the tail of H4 interacts with the N-terminal region of DOT1L. The H4 tail and ubiquitin clamp the opposing ends of DOT1L to position the DOT1L active site over H3K79 (Fig. 4D) (Worden et al., 2019). Remarkably, histone H3 also undergoes a conformational change to reorient K79^Nle^ outwards, thus enabling access of H3K79^Nle^ into DOT1L’s active site (Fig. 4D) (Worden et al., 2019).

In addition to H2BK120ub, the activity of DOT1L can also be regulated by H4K16ac (Lee et al., 2018b; Valencia-Sánchez et al., 2021). The cryo-EM structure of yeast Dot1 in complex with the H4K16ac-H2BK123ub nucleosome provides a plausible explanation for Dot1 stimulation by H4K16ac (Valencia-Sánchez et al., 2021). Unlike the unacetylated NCP-bound structure that only has traceable H4 density from H4K16, the acetylation of H4K16 enables clear visualization of the H4 tail (G11–K16), including the side-chain of H4K16ac (Fig. 4E) (Valencia-Sánchez et al., 2021). The acetyl group of H4K16ac establishes multiple interactions with residues in the acetyl-gate loop (“AcG loop”; residues 252–264) of Dot1, which can further result in the overall stabilization of the AcG loop and the H4 N-terminal tail (Fig. 4E) (Valencia-Sánchez et al., 2021).

### H4K20 methyltransferases

SET8 (also known as KMT5A, PR-SET7, or SETD8) is the only known methyltransferase responsible for H4K20 monomethylation and plays pivotal roles in genome replication and stability (Fang et al., 2002; Nishioka et al., 2002; Jørgensen et al., 2007; Oda et al., 2009). The crystal structures of the SET8 SET domain with a histone H4 peptide have provided insights into the catalytic mechanism of SET8 acting on the H4 peptide (Couture et al., 2005, 2008; Xiao et al., 2005). However, SET8 exhibits significantly greater methyltransferase activity on nucleosomes than on histone peptides (Fang et al., 2002; Nishioka et al., 2002), indicating an additional regulatory mechanism upon binding with nucleosomes. The cryo-EM structures of SET8-NCP and SET8-NCP^CENP-A^ complexes reveal that SET8 binds to the disc surface of the histone core, with no apparent contact with the nucleosomal DNA (Ho et al., 2021). SET8 specifically recognizes the nucleosomal acidic patch via an arginine anchor formed by R188 and R192 in the N-terminal extension of the SET8 SET domain, thus dictating the SET domain orientation on the nucleosome to accommodate the H4 N-terminal tail in the active pocket (Ho et al., 2021). Mutagenesis analyses confirm the importance of the interaction between arginine anchor and histone acidic patch in nucleosome binding and H4K20 monomethylation, explaining why SET8 prefers the nucleosome substrate over the H4 peptide.

### HKMT mutations in cancers

Numerous studies have shown that mutations or dysregulations of HKMTs can lead to physiological abnormalities and cause a wide range of human diseases (Table 2) (Bhat et al., 2021; Zhao et al., 2021). Accordingly, HKMTs have been considered as attractive therapeutic targets for diverse diseases. Over the past decade, many HKMT inhibitors have been developed and are in different phases of clinical trials or preclinical studies (Bhat et al., 2021). Most HKMT inhibitors developed to date target the lysine substrate (lysine) or cofactor (AdoMet) binding site. Recent structural studies of HKMT nucleosomes provide new insights into the impact of cancer-associated mutations and offer alternative strategies to target the newly identified HKMT-nucleosome interfaces. Here, we briefly review the pathological features of HKMTs and propose some potential nucleosome-dependent targets for further drug development.

**Table 2.** HKMT dysregulation and mutations in cancers.

Methylation site	Genes	Mutations in cancer	Putative roles in cancer
H3K4	*MLL1*	*MLL1* rearrangements in AML, ALL or MLL (Krivtsov and Armstrong, 2007); overexpression in in p53 GOF patient-derived tumors (Zhu et al., 2015)	Oncogenic
	*MLL2*	Overexpression inbreast and colorectal cancers (Natarajan et al., 2010)	Oncogenic
	*MLL3*	Loss-of-function mutations in skin, urothelial, bladder, lung, head and neck, esophagogastric, colorectal, small bowel cancers (Fagan and Dingwall, 2019), andnon-Hodgkin lymphoma (MLL4 R5432W) (Li et al., 2016)	Tumor suppressive
	*MLL4*		Tumor suppressive
H3K27	*EZH2*	Gain-of-function mutations (Y641X) in DLBCL and follicular lymphomas; Overexpression in prostate, breast, bladder, endometrial and melanoma cancers; loss-of-function mutations in MDS, MPN, T-ALL (Kim and Roberts, 2016), melanoma (P132S, D142V), myelofibrosis (P132S), and Weaver Syndrome (P132S, F145L) (Lee et al., 2018a)	Oncogenic/tumor suppressive
	*EED*	Loss-of-function mutations in ETP ALL, MPNSTs, MDS (Kim and Roberts, 2016), Weaver Syndrome (H258Y, R302G, R302S), ALL (S259F) and MPN (R302G) (Lee et al., 2018a)	
	*SUZ12*	Loss-of-function mutations in T-ALL, ETP ALL, MPNSTs (Kim and Roberts, 2016)	
H3K36	*SETD2*	Loss-of-function mutations in renal-cell carcinoma, lung adenocarcinoma, hematological malignancies, central nervous system tumors, bladder cancer, and gastrointestinal tumors (Husmann and Gozani, 2019)	Tumor suppressive
	*NSD2*	Gain-of-function mutations: E1099K inALL, lung adenocarcinoma, colon cancer, thyroid tumors (Husmann and Gozani, 2019), and T1150A in mantle cell lymphoma (Beà et al., 2013); overexpression in multiple myeloma and prostate cancer (Husmann and Gozani, 2019)	Oncogenic
	*NSD3*	Gain-of-function mutations: E1181K and T1232A (Li et al., 2021a); overexpression in breast cancer, LSCC and SCCHN (Husmann and Gozani, 2019)	Oncogenic
H3K79	*DOT1L*	Overexpression in breast and prostate cancers (Nassa et al., 2019; Vatapalli et al., 2020)	Oncogenic

### H3K4/H3K79 methyltransferases in cancers

Mixed lineage leukemia (MLL) is a malignant hematological tumor with high incidence and poor prognosis and is caused by rearrangements in the allele of *MLL1* (Hilden et al., 2006; Krivtsov and Armstrong, 2007). Mechanistically, with a break between the CXXC and PHD domains, the N-terminal portion of MLL1 can fuse with more than 70 different fusion partners, many of which can recruit the super elongation complex and the H3K79 methyltransferase DOT1L (Krivtsov et al., 2017). The remaining wild-type copy of MLL1 and its methyltransferase activity are also required for MLL1 fusion protein-induced leukemogenesis (Thiel et al., 2010; Cao et al., 2014). Moreover, there is increasing evidence that MLL1 functions as an oncogene in many other cancers (Ansari et al., 2013; Zhu et al., 2015, 2019). MLL2, the closest paralog of MLL1, is overexpressed in breast and colorectal cancers (Natarajan et al., 2010), and MLL2 also plays a significant role in sustaining *MLL*-rearranged leukemia (Chen et al., 2017). Unlike MLL1/2, MLL3 and MLL4 often function as tumor suppressors. *MLL3* and *MLL4* are among the most frequently mutated epigenetic genes associated with human cancers, and mutations involving *MLL3*/*4* often result in reduced protein functions through missense alterations or truncations (Fagan and Dingwall, 2019). For example, the MLL4 R5432W mutation identified in non-Hodgkin lymphoma completely disrupts MLL4’s interaction with RBBP5-ASH2L and abolishes HKMT activity (Li et al., 2016). MLL3/4 inactivation results in a significant reduction of H3K4me1 on enhancers and provokes tumorigenesis (Zhang et al., 2015a; Alam et al., 2020; Larsson et al., 2020; Zheng et al., 2021).

**Figure 5.**
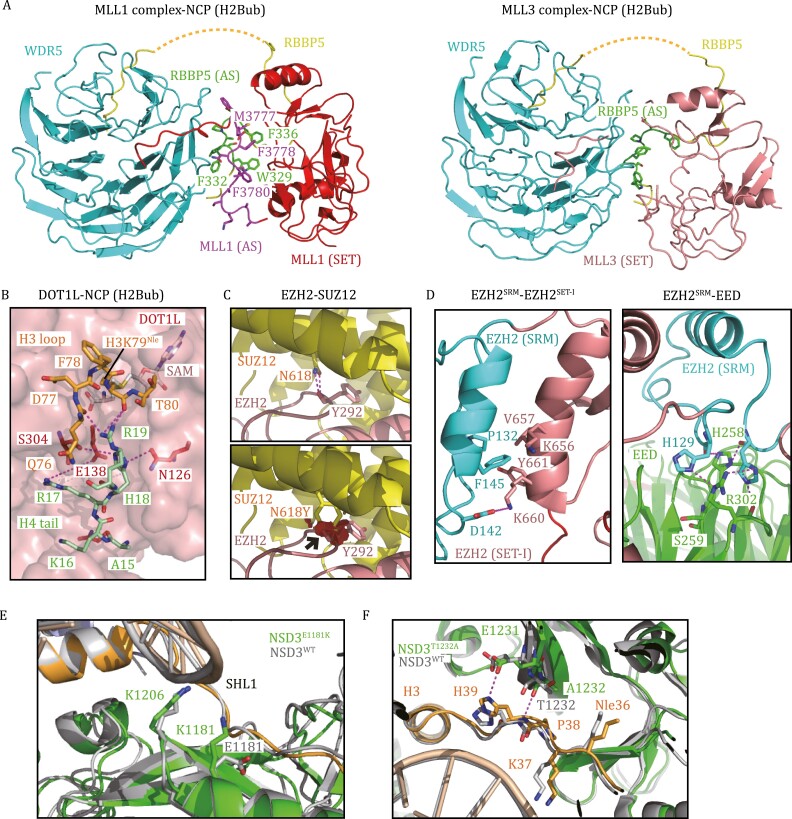
Histone lysine methyltransferases in human diseases. (A) The interface between WDR5, RBBP5, and MLL1/3 (PDB: 6KIU, 6KIW). RBBP5_AS_ is colored green, and MLL1_AS_ is colored purple. Residues that mediate hydrophobic interactions between the interfaces of RBBP5_AS_ and MLL1_AS_ are highlighted. (B) A stretch of basic residues in the tail of H4 binds DOT1L and the H3K79 loop (PDB: 6NQA). (C) The EZH2–SUZ12 interface around SUZ12^N618^ (PDB: 5HYN). The SUZ12^N618Y^ mutation may introduce a steric clash between SUZ12^N618^ and EZH2^Y292^ (black arrow), as denoted by red plates. (D) The interfaces of EZH2^SRM^–EZH2^SET-I^ and EZH2^SRM^–EED (PDB: 5HYN). Caner-mutation-associated residues, which mediate protein–protein interactions, are highlighted as sticks. (E) Superposition of the structures of NSD3^WT^-NCP and NSD3^E1181K/T1232A^-NCP shows that E1181K increases the electrostatic interaction between NSD3 and NCP (PDB: 7CRR, 7CRP). (F) Superposition of the structures of NSD3^WT^-NCP and NSD3^E1181K/T1232A^-NCP shows that T1232A induces H3 to move closer to NSD3 (PDB: 7CRR, 7CRP).

Considering the multifaceted functions of MLL family proteins in cancers, the development of selective inhibitors to target their methyltransferase activity is highly desirable. Notably, the SET domains of MLL family proteins are highly conserved and show nearly identical binding patterns with substrates (Zhang et al., 2015b; Li et al., 2016, 2020), highlighting the challenges in designing selective inhibitors targeting the active sites of these highly homologous enzymes. To date, all MLL1 inhibitors have been rationally designed based on structural studies by targeting critical protein–protein interactions in the MLL1 complex, including the MENIN–MLL1 interaction and WDR5–MLL1 interaction (Li et al., 2021b). Recent structural studies may provide more potential strategies for future drug development. For example, Xue et al., (2019) demonstrated that the MLL1–ubNCP complex and MLL3–ubNCP complex display different structural organizations at the interface between WDR5, RBBP5, and MLL1/3 (Fig. 5A). The activation segment of MLL1 (MLL1_AS_: 3775–3786), which is not conserved among MLL family proteins, extends along the β-propeller surface of WDR5 and interacts with the activation segment of RBBP5 (RBBP5_AS_: 329–336) through an extensive network of hydrophobic interactions (Fig. 5A) (Xue et al., 2019). Deleting both MLL1_AS_ and RBBP5_AS_ resulted in a 75% loss of MLL1 activity, indicating that specific inhibitors targeting this MLL1-unique interface could be feasible. Conversely, WDR5 makes direct contact with MLL3_SET_ and significantly represses the activities of MLL3 on nucleosomes (Fig. 5A) (Xue et al., 2019). Targeting the WDR5–MLL3_SET_ interface may specifically stimulate MLL3 activity.

Notably, the HMTase activity of DOT1L is an important driver in most *MLL*-rearranged leukemias (Okada et al., 2005; Bernt et al., 2011; Deshpande et al., 2013). AdoMet-competitive DOT1L inhibitors selectively kill mixed lineage leukemia cells, prolong survival in a mouse xenograft model of *MLL*-rearranged leukemia and have been in clinical trials (e.g. Pinometostat) (Daigle et al., 2011, 2013). In addition, DOT1L is also a promising therapeutic target in breast and prostate cancers (Nassa et al., 2019; Vatapalli et al., 2020). Structural studies of DOT1L in complex with nucleosomes suggest alternative strategies for designing specific DOT1L inhibitors. For example, DOT1L establishes multivalent interactions on the surface of nucleosomes, and disrupting the binding of DOT1L to ubiquitin or the acidic patch can significantly reduce its enzymatic activity (Valencia-Sánchez et al., 2019; Worden et al., 2019). Furthermore, a stretch of basic residues in the tail of H4 (_16_KRHR_19_) binds to a groove in the N-terminal portion of DOT1L and is also essential for the HKMT activity of DOT1L (Fig. 5B) (Worden et al., 2019). The H4 tail binding groove is contiguous with the active site tunnel. Specific inhibitors that bind to these DOT1L-nucleosome interfaces could more effectively decrease the HKMT activity of DOT1L (Fig. 5B) (Worden et al., 2019).

### H3K27 methyltransferases in cancers

EZH2 is overexpressed in a wide range of cancer types (Kim et al., 2016). The “hotspot” gain-of-function mutations at Y641 (Y641X, X = F, N, C, S, or H) within the catalytic domain of EZH2 were found in 22% of patients with GCB-type diffuse large B-cell lymphoma (DLBCL) and 7% of patients with follicular lymphomas (Morin et al., 2010; Yap et al., 2011). Mechanistically, EZH2 Y641X mutations reduce steric crowding in the active site and alter the product specificity of EZH2, leading to enhanced H3K27me3 levels (Sneeringer et al., 2010; Yap et al., 2011). AdoMet-competitive inhibitors targeting EZH2 represent an attractive anticancer strategy, with Tazemetostat becoming the first-in-class FDA-approved HKMT-inhibitory drug in 2020 (Rothbart and Baylin, 2020). To date, Tazemetostat has been approved for the treatment of epithelioid sarcoma and relapsed or refractory follicular lymphoma (Rothbart and Baylin, 2020). Tazemetostat has also been used in various stages of clinical trials for a variety of other diseases, with promising interim data (Bhat et al., 2021).

On the other hand, PRC2 can function as a tumor suppressor. Loss-of-function mutations in EZH2, EED, or SUZ12 occur in a subset of myeloid malignancies, T-cell acute lymphoblastic leukemia (T-ALL), and malignant peripheral nerve sheath tumors (MPNSTs) (Kim and Roberts, 2016). Structural and functional studies of PRC2 reveal that missense mutations preferentially occur in the CXC-SET domain of EZH2 or the assembly interface of the PRC2 complex, leading to reduced PRC2 activity (Ernst et al., 2010; Brooun et al., 2016; Lee et al., 2018a). For example, SUZ12^N618^ is buried in the SUZ12–EZH2 interface and forms hydrogen bonds with EZH2^Y292^ (Fig. 5C) (Brooun et al., 2016). A patient-derived SUZ12^N618Y^ mutation may introduce steric clashes and compromise the integrity of the PRC2 complex (Fig. 5C) (Brooun et al., 2016). In addition, several EZH2/EED mutations located at the interfaces of EZH2^SRM^–EZH2^SET-I^ and EZH2^SRM^–EED abrogate allosteric activation of PRC2, including P132S/D142V/F145L of EZH2 and H258Y/S259F/R302S/R302G of EED (Fig. 5D) (Lee et al., 2018a). Specifically, disrupting PRC2 allosteric activation can override the hyperactivity of EZH2^Y641X^ and inhibit the proliferation of PRC2/EZH2-addicted tumors, providing a potential therapeutic strategy for PRC2/EZH2-addicted cancers by targeting EZH2^SRM^–EZH2^SET-I^ and EZH2^SRM^–EED interactions (Lee et al., 2018a).

### H3K36 methyltransferases in cancers

The H3K36me3 methyltransferase SETD2 is generally proposed to function as a tumor suppressor, and loss-of-function mutations in SETD2 occur in a broad spectrum of human malignancies (Duns et al., 2010; Husmann and Gozani, 2019). In contrast, H3K36me2-specific methyltransferases, including NSD1, NSD2, NSD3, and ASH1L, are commonly thought to promote oncogenesis (Husmann and Gozani, 2019). Remarkably, the “hotspot” E1099K mutation within the catalytic domain of NSD2 is found in 14% of pediatric ALLs (Jaffe et al., 2013) and other neoplasms, such as lung adenocarcinoma, colon cancer, and thyroid tumors (Imielinski et al., 2012; Husmann and Gozani, 2019). Another NSD2_SET_ mutation, T1150A, is also recurrently found in mantle cell lymphoma (Beà et al., 2013). All NSD2^E1099K^, NSD2^T1150A^, and NSD2^E1099K/T1150A^ mutations can enhance the HMTase activity of NSD2 (Li et al., 2021a). Similarly, the equivalent E1181K and T1232A substitutions in NSD3 have also been linked with cancer and render NSD3 a more active enzyme (Li et al., 2021a). The cryo-EM structures of NSD2^E1099K/T1150A^/NSD3^E1181K/T1232A^/NSD3^WT^ in complex with 187-bp NCPs reveal the molecular mechanisms of NSD2/NSD3 activity regulation by these two mutations (Li et al., 2021a). Specifically, E1181 of NSD3 is positioned close to SHL1 DNA, and replacing a negatively charged Glu with a positively charged Lys can increase the electrostatic interactions between NSD3 and the nucleosome (Fig. 5E) (Li et al., 2021a). The side-chain methyl group of NSD3^T1232^ is inserted into a hydrophobic pocket surrounded by four hydrophobic residues of NSD3 and H3^P38^. The replacement with a smaller alanine side-chain could induce the main chains of H3 P38-H39 to move closer to NSD3 and allow the formation of new interactions between NSD3^T1232A/E1231^ and H3^H39^ (Fig. 5F) (Li et al., 2021a). Thus, both mutations can enhance the enzymatic activity of NSD3. Similar mechanisms might also apply to the E1099K and T1150A mutations in NSD2, providing critical insights into the role of NSD2/3 in driving tumor progression.

## Concluding remarks

The growing number of structural studies of HKMTs bound to nucleosomes has provided valuable insights into the molecular mechanism of nucleosome recognition and catalysis by HKMTs. As of this writing, only the structures of H3K9 methyltransferases with nucleosomes remain unresolved. H3K9 methylation is a hallmark of heterochromatin and is an important modification for diverse cellular processes (Padeken et al., 2022). Recent studies have discovered new binding sites between H3K9 methyltransferases and nucleosomes (Akoury et al., 2019). For example, the disordered region between the chromo domain and SET domain of Clr4, an H3K9 methyltransferase in fission yeast, binds to the nucleosome core and plays an important role in H3K9 methylation (Akoury et al., 2019). We expect the structures of H3K9 methyltransferases in complex with nucleosomes to be revealed in the near future, providing insights into heterochromatin formation and spreading by H3K9 methyltransferases.

The crosstalk mechanisms between histone ubiquitination (H2B K120ub) and histone methylation (H3K4me3, H3K36me3, H3K79me3) have been elucidated. It is expected that future biochemical and structural studies will uncover more crosstalk between a specific histone methylation and other histone modifications from the same nucleosome and the neighboring nucleosome, highlighting how the “histone code” is synergistically established and interpreted by a combination of histone-modifying enzymes.

The H2A–H2B acidic patch creates a highly negatively charged groove on nucleosomes and is a docking “hotspot” for most chromatin-binding proteins, including histone-modifying enzymes, chromatin remodeling complexes, and polymerase complexes (Fig. 1J). Most HKMTs can recognize the “acidic patch” through an arginine anchor containing several arginine residues. This specific recognition ensures proper nucleosome binding and catalytic activity. However, not all methyltransferases bind to the acidic patch. For instance, COMPASS interacts with the acidic patch through the ARM helix of Set1, but the homologous MLL1 complex does not have any motif recognizing the acidic patch. Additionally, the H3K36-family methyltransferases do not appear to bind the H2A–H2B acidic patch. These outliers suggest the diversity and complexity in nucleosome recognition and regulation by different HKMTs.

It should be noted that the current HKMT-nucleosome structures are all determined at the mononucleosomal or dinucleosomal levels. It will now be essential to extend similar structural studies to nucleosome arrays or chromatin fibers, which are the more physiological substrates for HKMTs. The high-resolution structures of tetranucleosomes and 30 nm chromatin fibers reveal that, in folded chromatin fibers, the nucleosomal H2A–H2B acidic patch is occupied by the H4 tail from the neighboring nucleosome, and this interaction is vital for chromatin compaction (Schalch et al., 2005; Song et al., 2014). Considering that the H2A–H2B acidic patch is the common anchoring site for most HKMTs, it is intriguing to see how HKMTs recognize, methylate, and potentially remodel chromatin fibers.

HKMTs often orchestrate transcription factors, histone chaperones, chromatin remodeling complexes, and non-coding RNAs to regulate nucleosome structure and downstream transcription. How HKMTs collaborate with other factors to establish the epigenetic states of chromatin remains largely unexplored. For example, a recent report found that the MLL-family methyltransferase MLL4 associates with the chromatin remodeling complex BAF (Park et al., 2021), but how these two complexes couple together to regulate enhancer activation is unknown. There is also mounting evidence that HKMTs can interact with histone chaperones and histone variants to modulate chromatin structure, regulate gene expression, and maintain genome stability (Sarai et al., 2013; Li et al., 2017; Higgs et al., 2018; Ouda et al., 2018), but how HKMTs coordinate with histone chaperones remains elusive. Thus, structural studies of HKMTs in complexes with other epigenetic factors (e.g. chromatin remodeling complexes, histone chaperones, histone variants, and non-coding RNAs) will be the next frontier to reveal how these complexes act cooperatively on nucleosomes or chromatin substrates.

Growing evidence has demonstrated the close association between the misregulation of HKMTs and tumorigenesis (Table 2), so targeting the enzymatic activity of HKMT has emerged as an important pathway for cancer therapy (Bhat et al., 2021). Structural studies of HKMTs in complex nucleosomes demonstrate diverse binding modes with nucleosomes, enabling us to design specific inhibitors for each HKMT that target its unique structural element for nucleosome recognition and catalysis. This could contribute to the discovery of novel therapeutic drugs for cancer treatment.

## Abbreviations

AcG loop: acetyl-gate loop
AdoMet: *S*-adenosylmethionine
AEBP2: adipocyte enhancer-binding protein 2
ARM: arginine-rich motif
AS: activation segment
ASH2L: absent: small or homeotic 2-like protein
AWS: associated with SET
BAF: BRG1/BRM-associated factor
CENP-A: centromere protein A
COMPASS: complex proteins associated with Set1
C2H2: Cys2-His2
cryo-EM: cryo-electron microscopy
DLBCL: diffuse large B-cell lymphoma
DNA: deoxyribonucleic acid
Dot1: disruptor of telomere silencing 1
DOT1L: Dot1-like
DPY30: dumpy-30
EED: embryonic ectoderm development
EPOP: elongin BC and polycomb repressive complex 2-associated protein
ETP ALL: early T-cell precursor acute lymphoblastic leukemia
EZH1/2: enhancer-of-zeste homolog 1/2
FDA: food and drug administration
GCB: germinal center B-cell-like
H2A: histone H2A
H2B: histone H2B
H3: histone H3
H4: histone H4
H3K4: histone H3 lysine 4
H3K9: histone H3 lysine 9
H3K27: histone H3 lysine 27
H3K36: histone H3 lysine 36
H3K79: histone H3 lysine 79
H4K20: histone H4 lysine 20
HKMT: histone lysine methyltransferase
JARID2: jumonji/AT-rich interaction domain-containing 2
KMT2: histone lysine *N*-methyltransferase 2
LSCC: lung squamous cell carcinoma
MCSS: motif connecting SANT1L and SANT2L
MDS: myelodysplastic syndrome
MLL: mixed lineage leukemia
MPN: myeloproliferative neoplasm
MPNST: malignant peripheral nerve sheath tumor
MS2L: MCSS/SANT2L loop
MTF2: metal-response element-binding transcription factor 2
NCP: nucleosome core particle
Nle: norleucine
NSD1/2/3: nuclear receptor-binding SET domain-containing protein 1/2/3
PALI1/2: PRC2 associated LCOR isoform 1/2
PHD: plant homeodomain
PHF1: PHD finger protein 1
PHF19: PHD finger protein 19
PRC2: polycomb repressive complex 2
PTM: posttranslational modification
RBAP46/48: retinoblastoma-associated protein 46/48
RBBP5: retinoblastoma-binding protein 5
RNA: ribonucleic acid
SBD: SANT-binding domain
SCCHN: squamous cell carcinoma of the head and neck
SET: su(var)3-9: enhancer-of-zeste: and trithorax
SET8: SET domain-containing protein 8
SHL: superhelix
SPRY: spla/ryanodine receptor
SRM: stimulation-responsive motif
SUZ12: suppressor of zeste 12
T-ALL: T-cell acute lymphoblastic leukemia
UIM: ubiquitin interacting motif
WDR5: WD repeat-containing protein 5

## Declarations

This work was supported by grants from the Strategic Priority Research Program of the Chinese Academy of Sciences (XDB37010303 to Y.C.), the National Natural Science Foundation of China (31670748, 31970576 to Y.C., and 32071195 and 31900934 to Y.L.), the Young Elite Scientist Sponsorship Program by Chinese Association for Science and Technology (YESS20170198 to Y.L.), and the National Postdoctoral Program for Innovative Talents (BX201700263 to Y.L.). We apologize to all those whose work we have been unable to include due to space limitations.

Y.L. and Y.C. discussed the structure and organization of the manuscript. Y.L., K.G., T.L., R.C. and Y.C. wrote the manuscript.

This article does not contain any studies with human or animal subjects performed by the any of the authors.
